# The Potential Use of Cannabidiol in the Treatment of Opioid Use Disorder: A Systematic Review

**DOI:** 10.1111/adb.70047

**Published:** 2025-05-25

**Authors:** Mahan Shafie, Kevin Ing, Yasna Rostam‐Abadi, Jeremy Weleff, Mackenzie Griffin, Mohini Ranganathan, Ardavan Mohammad Aghaei, Nicholas Pratt, Melissa C. Funaro, Anahita Bassir Nia

**Affiliations:** ^1^ School of Medicine Tehran University of Medical Sciences Tehran Iran; ^2^ Department of Psychiatry University of California San Diego La Jolla California USA; ^3^ VA San Diego Healthcare System San Diego California USA; ^4^ Department of Psychiatry Yale University School of Medicine New Haven Connecticut USA; ^5^ Department of Psychiatry, Faculty of Medicine & Dentistry University of Alberta Edmonton Alberta Canada; ^6^ Neuroscience and Mental Health Institute University of Alberta Edmonton Alberta Canada; ^7^ VA Connecticut Healthcare System West Haven Connecticut USA; ^8^ Harvey Cushing/John Hay Whitney Medical Library Yale University New Haven Connecticut USA; ^9^ Clinical Neurosciences Division National Center for PTSD (NCPTD), VA Connecticut Healthcare System West Haven Connecticut USA

**Keywords:** addiction, cannabidiol, opioid, opioid use disorder, tetrahydrocannabinol, withdrawal

## Abstract

Cannabidiol (CBD) has emerged as a potential treatment option for various psychiatric disorders, including substance use disorders. This systematic review is aimed at reviewing the evidence regarding the safety and efficacy of CBD as a therapeutic option in opioid use disorder (OUD) treatment in clinical and preclinical studies. We searched MEDLINE, Embase, PsycINFO, Scopus, Web of Science, CDSR and CENTRAL up to December 2023. We included original peer‐reviewed human and animal studies evaluating CBD for OUD outcomes and excluded those that did not report OUD outcomes or used CBD solely with THC. The risk of bias was assessed with the Cochrane risk‐of‐bias tool for human studies and SYRCLE's tool for animal studies. Due to outcome heterogeneity, findings were presented using a qualitative synthesis. Four clinical studies (74 participants) and 16 preclinical studies met the inclusion criteria. The collective evidence from clinical and preclinical studies indicates that CBD holds promise as an adjunctive therapy for OUD with a well‐tolerated profile during opioid use and withdrawal. Human clinical studies demonstrated a reduction in craving and alleviation of abstinence‐induced anxiety. In preclinical studies, CBD has been shown to reduce withdrawal symptoms and diminish opioid‐rewarding effects using the conditioned place preference paradigm, although the results are mixed, and not all preclinical studies reported these effects. The quality assessment for clinical studies indicated an overall evaluation of ‘some concerns’, while a notable level of ‘unclear’ risk was observed across the evaluated domains for preclinical studies. This systematic review highlights the potential of CBD as a beneficial treatment option for addressing cravings and anxiety symptoms during abstinence in individuals with OUD, based on findings from human studies. Continued research and clinical trials will be essential for further improving outcomes in OUD treatment using novel effective treatment approaches. Study limitations include the limited number of clinical studies, small sample size, short‐term follow‐up, lack of combination therapy and heterogeneity across preclinical studies.

**Trial Registration:**

PROSPERO identifier: CRD42023401446

## Graphical abstract

This systematic review found that CBD may reduce opioid cravings and abstinence‐related anxiety in individuals with opioid use disorder. Preclinical studies also suggest CBD can reduce withdrawal symptoms, opioid‐rewarding effects, relapse, and neuroinflammation. Together, these findings support CBD's potential as an adjunctive treatment for OUD, though clinical evidence remains limited.

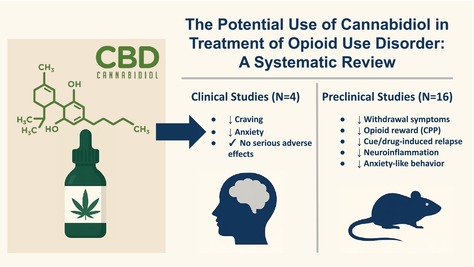

## 1 Introduction

Opioid use disorder (OUD) has become an escalating global problem, with significant morbidity and mortality affecting more than 20 million people worldwide, resulting in more than 80 000 opioid‐related deaths and approximately 13 000 person‐year disability‐adjusted life years in 2019 [1]. The economic burden of OUD exceeded a trillion dollars in 2017, primarily driven by reduced quality of life and the value of lives lost to fatal opioid overdoses [2]. While medications such as buprenorphine or methadone have proven effective in reducing opioid use, increasing treatment retention and reducing all‐cause mortality, a major challenge remains in ensuring broader access and retention, with many patients still experiencing relapse and prematurely discontinuing treatment [3, 4]. Despite their proven efficacy, only about 25% of individuals with OUD in the United States received medication for OUD, and treatment retention remains suboptimal [5]. Furthermore, opioid substitution therapy primarily targets the mu‐opioid receptor (MOR), leading to receiving long‐term maintenance opioid [6]. Given the lack of established augmentation options for OUD treatment, there is an urgent need to identify novel and potentially effective treatment approaches for individuals with OUD.

The endocannabinoid (eCB) system has emerged as a significant focus of interest due to its intricate interplay with the endogenous opioid system [7]. Both the cannabinoid receptor type 1 (CB1R) and the opioid MOR are Gi/o‐coupled receptors, sharing anatomical and functional characteristics [8], which result in overlapping behavioural effects, such as sedation, analgesia and reward perception [9, 10]. Research has highlighted the synergistic effects and cross‐tolerance between cannabinoids and opioids [11, 12, 13]. Anatomically, a bidirectional modulation of their rewarding and reinforcing properties has been observed in key brain regions involved in addiction, including the ventral tegmental area (VTA), nucleus accumbens (NAc) and basal ganglia [14, 15, 16]. This dynamic interaction between the endogenous opioid system and the eCB system offers potential therapeutic avenues for targeting the eCB system in treating OUD [17]. Additionally, as a major stress regulatory network, the eCB system may significantly influence stress‐induced opioid craving and relapse in OUD [18]. The eCB system also interacts with other neural circuits, including dopamine, gamma‐aminobutyric acid and glutamate systems, that are involved in cognitive processes related to reward processing, emotional regulation and stress response [19, 20, 21]. Therefore, further exploration of targeting the eCB system in OUD treatment is crucial for developing potential novel effective therapeutic strategies for OUD.

Cannabinoids such as cannabidiol (CBD) and delta‐9‐tetrahydrocannabinol (THC) interact with the eCB system in diverse ways [22, 23, 24]. The two primary receptors in the eCB system are CB1R and cannabinoid receptor type 2 (CB2R), which bind to the main endogenous eCB ligands, anandamide (AEA) and 2‐arachidonoylglycerol (2AG) [18]. THC, the primary psychoactive component of cannabis, acts as a partial agonist at these receptors [25], while CBD, a nonaddictive and nonpsychoactive component of cannabis, activates other targets, though its exact mechanism of action is still being explored [26]. Specifically, CBD, with lower affinity for CB1/2R, indirectly modulates eCB system activity by inhibiting fatty acid amide hydrolase (FAAH), AEA degrading enzyme, leading to increased AEA levels. Other proposed mechanisms of action for CBD include interacting with TRPV1 for pain regulation, GPR55 for neuromodulation and PPARγ for anti‐inflammatory effects [27].

Growing evidence over recent decades suggests that targeting the eCB system in treating OUD is promising. Several preclinical studies have demonstrated that the modulation of the eCB system can reduce opioid‐seeking and opioid withdrawal behaviours [28, 29]. Similarly, human studies have reported that the eCB system modulators are effective in attenuating opioid withdrawal symptoms, craving and reinforcing effects of opioids [30, 31, 32]. Although research on the effects of cannabis on OUD treatment remains inconclusive [33], emerging evidence highlights the potential therapeutic use of CBD, which is already FDA‐approved for treating refractory seizure disorders, with safety and tolerability demonstrated in numerous trials [34]. Furthermore, several studies have reported CBD's anxiolytic effects in clinical and preclinical studies [22, 23, 24]. Given the crosstalk between the eCB and endogenous opioid system, the eCB system's involvement in drug‐seeking behaviours, its central role in stress regulation and CBD's reported safety and tolerability, it is imperative to understand its therapeutic potential in OUD [35]. This systematic review summarizes the available evidence for CBD's potential effectiveness in treating OUD, focusing on its effectiveness in reducing cravings, alleviating anxiety and controlling withdrawal symptoms, particularly in the abstinence and recovery phases of OUD treatment.

## 2 Methods

### 2.1 Search Strategy

We adhered to the PRISMA guidelines checklist to report the findings of this systematic review. A health sciences librarian specializing in search strategy development for systematic reviews in the mental health field crafted the search strategies. The initial search strategy was created with inputs from the research team and subsequently peer‐reviewed by a second librarian, who was not otherwise associated with the project, using the PRESS standard. Our search strategy focused on two main key terms: (1) terms related to CBD and (2) terms related to OUD. Detailed search strategies can be found in Table S1. The search was performed on November 17, 2022, and subsequently updated on December 4, 2023, to include the most recent and relevant studies.

### 2.2 Information Sources

We conducted searches in major online international scientific databases, including MEDLINE, Embase, PsycINFO, Scopus, Web of Science, Cochrane Database of Systematic Reviews (CDSR) and Cochrane Central Register of Controlled Trials (CENTRAL). To ensure literature saturation, we scanned the reference lists of included studies and reviews identified through the search (backward citation tracking). The electronic database search was supplemented by searching the Clinical Trials Registry Platform Search Portal and ClinicalTrials.gov. Additionally, we searched PROSPERO for any relevant ongoing or recently completed systematic reviews. Where necessary, we sought additional data from study authors to address questions about eligibility.

### 2.3 Eligibility Criteria

We included full‐length original human and animal studies published in peer‐reviewed journals that evaluated the effects of CBD on OUD, encompassing randomized controlled trials, controlled clinical trials, cluster trials, cohort studies, case‐control studies, cross‐sectional studies, case series, case reports and preclinical studies. We excluded studies that either (1) did not report any outcomes related to OUD, focusing instead on other outcomes such as pain management, anxiety or epilepsy, or (2) used CBD only in combination with THC.

### 2.4 Data Management

Literature search results were uploaded to EndNote [36] and deduplicated using the Reference Deduplicator [37]. More duplicates were found after uploading this set to Covidence, an internet‐based software program that facilitates reviewers' collaboration during the study selection process.

### 2.5 Selection Process

The team developed screening questions based on eligibility criteria and adjusted them as needed after pilot screening the first 1569 records. Reviewers (K.I., Y.R.‐A., J.W., A.M.A. and N.P.) independently screened the titles and abstracts yielded by the search against the exclusion criteria. We obtained full reports for all titles that appeared to meet the inclusion criteria or where there was uncertainty. Pairs of review authors (M.S., K.I., Y.R.‐A., J.W., M.G., A.M.A. and N.P.) then screened the full‐text reports to determine whether they met the inclusion criteria. Disagreements were resolved through discussion or by consulting a third reviewer. A total of 101 disagreements occurred at the title and abstract screening and eight at the full‐text review stage.

### 2.6 Data Extraction

#### 2.6.1 Data Items

We extracted bibliographic information and publication status, trial design, trial size, the type of opioid, the generic and trade name of the CBD, the type of control, the route of administration and dosages and frequency and duration of treatment in each arm for all of the included studies. For clinical studies, patients' demographic characteristics, medication for OUD, OUD definition, outcome definition and outcome measures (adverse effects, withdrawal manifestations, cravings, relapse and standardized scales) were extracted. Animal models and animal behavioural outcomes, including withdrawal manifestations, conditioned place preference (CPP), drug‐seeking behaviours and other experimental paradigms, were also extracted from preclinical studies. Due to the heterogeneity of the outcomes, the summary findings of the included studies were presented using a qualitative synthesis, without further data synthesis.

#### 2.6.2 Quality Assessment/Risk of Bias (ROB)

We assessed human studies' ROB using the Cochrane ROB tool for clinical studies, which covers sequence generation, allocation concealment, blinding, incomplete outcome data and selective outcome reporting. For animal studies, we used SYRCLE's ROB tool. Two review authors (M.S., M.G. and A.M.A.) independently made these assessments. There were a total of six disagreements during ROB, which were resolved through discussion by consulting a third author for arbitration.

## 3 Results

**FIGURE 1:**
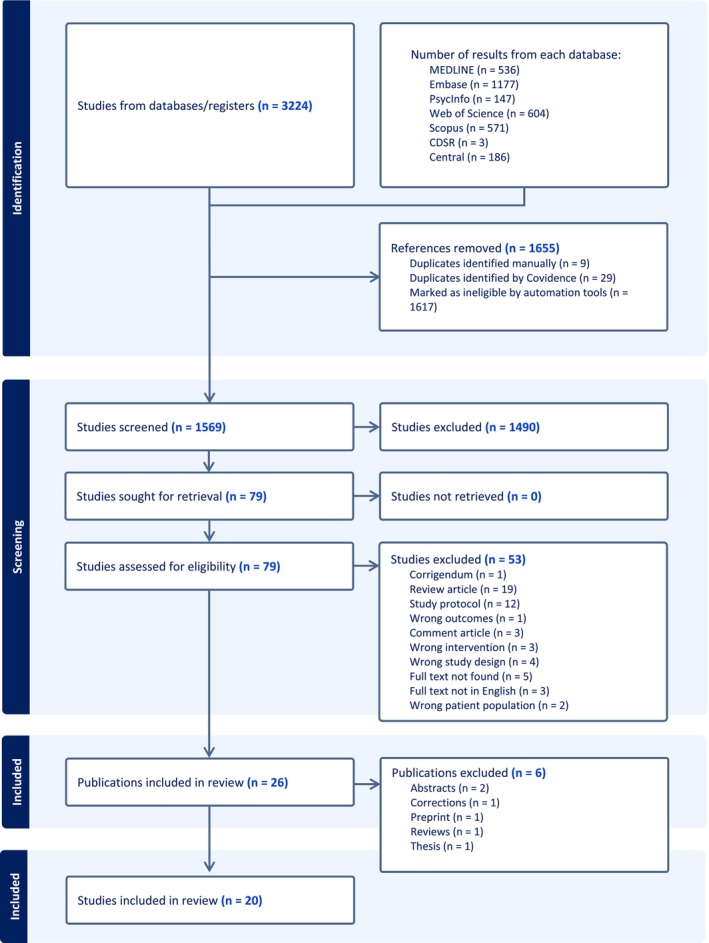
PRISMA flow diagram.

Our search resulted in 3224 papers (1569 after removing the duplications), and 79 were selected for full‐text reading (Figure 1). Ultimately, the eligibility criteria were met by four clinical and 16 preclinical studies.

### 3.1 Clinical Studies

This systematic review identified four clinical studies: three investigating the use of CBD in the treatment of OUD and one examining the coadministration of CBD with opioids in healthy subjects. The studies were published between 2015 and 2022 and consisted of three double‐blind, randomized, placebo‐controlled trials and one single‐arm open‐label pilot trial. The studies evaluated different doses of CBD, ranging from 400 to 800 mg (Table 1).

**TABLE 1:** Characteristics of reviewed clinical studies.

No.	Study/country	Trial design	Sample size	Sample characteristics	CBD dosage	Frequency and duration of treatment	Study period	Primary outcome measures	Secondary outcome measures	Outcome results
1	Hurd et al. (2019)/United States	Double‐blind randomized placebo‐controlled trial	42	Heroin‐abstinent individuals (most had been abstinent from heroin use for less than 1 month) Age: 49.8 (9.2) [21–65] Female: 16.7%	400 mg (*N* = 14) 800 mg (*N* = 13) Placebo (*N* = 15)	Once daily for 3 consecutive days	Two weeks	VAS‐C, out‐of‐clinic heroin craving questionnaire, VAS‐A	PANAS, cognition: Digit symbol substitution task, digit span test–backward, continuous performance task, physiological status: HR, T, BP, salivary cortisol level, adverse events	CBD reduced cue‐induced craving and anxiety, CBD reduced these measures 7 days after the final CBD exposure, CBD reduced physiological measures of HR and salivary cortisol levels, no significant effects on cognition, no serious adverse effects
2	Suzuki et al. (2022)/United States	Single‐arm open‐label pilot trial	5	Individuals with OUD receiving treatment with buprenorphine Age: 37.8 (7.8) Female: 20%	600 mg (*N* = 5)	Once daily for 3 consecutive days	Three days between two sessions	VAS‐C	PHQ‐9, GAD7, BPI, PANAS, COWS, craving (pre‐cue, post‐cue, neutral cue)	CBD reduced cue‐induced craving, with no significant changes in scores for depression, anxiety, pain or opioid withdrawal
3	Suzuki et al. (2023)/United States	Double‐blind, placebo‐controlled, cross‐over pilot trial	10	Individuals with OUD receiving treatment with buprenorphine or methadone Age: 45.1 (9.1) Female: 50%	600 mg (*N* = 10)	Two sessions separated by at least 1 week	At least 1 week between two sessions	VAS‐C, visual probe task	HR, BP, PHQ‐9, GAD7, BPI, PANAS, COWS, MCQ, IGT, MTPT, salivary cortisol	CBD decreased cue‐induced craving and attentional bias toward drug‐related cues, with no significant changes in other measures
4	Manini et al. (2015)/United States	Double‐blind placebo‐controlled cross‐over trial	17	Healthy volunteers with prior opioid exposure Age: 38.5 (2.2) [21–65] Female: 47%	400 mg (*N* = 6) 800 mg (*N* = 6) Placebo (*N* = 5)	Two sessions separated by at least 1 week	At least 1 week between two sessions	SAFTEE	O‐VAS, PANAS, VAS‐A, physiological status: HR, T, BP, RR, O2 saturation, plasma and urinary CBD concentrations, plasma cortisol	CBD was well tolerated at doses up to 800 mg, with no significant pharmacokinetic changes without respiratory depression or cardiovascular

Abbreviations: BP: blood pressure, BPI: brief pain inventory, CBD: cannabidiol, COWS: clinical opioid withdrawal scale, GAD7: generalized anxiety disorder 7, HR: heart rate, IGT: Iowa gambling test, MCQ: Monetary Choice Questionnaire, MTPT: mirror tracing persistence task, NA: not applicable, NR: not reported, OUD: opioid use disorder, O‐VAS: opioid visual analogue scale, PANAS: participants' positive and negative affect, PHQ‐9: Patient Health Questionnaire, RR: respiratory rate, SAFTEE: systematic assessment for treatment emergent events, T: temperature, VAS‐A: visual analogue scale cue‐induced anxiety, VAS‐C: visual analogue scale cue‐induced craving.

#### 3.1.1 Clinical Outcomes for OUD Treatment

Hurd et al. [38] conducted a double‐blind, randomized, placebo‐controlled clinical trial to evaluate the potential therapeutic effects of CBD in individuals with OUD who were not receiving medication for OUD treatment. Participants received a 400 or 800 mg dose of CBD (once daily for 3 consecutive days). The results indicated that CBD administration significantly reduced cue‐induced craving, natural opioid craving and anxiety in study participants, suggesting its potential as a promising treatment option for OUD. Additionally, CBD was found to lower physiological measures, including heart rate and salivary cortisol levels. There were no significant effects on cognition, and no serious adverse effects were observed.

Suzuki et al. [39] investigated the effect of CBD on cue‐induced craving in individuals with OUD on buprenorphine treatment in a single‐arm open‐label pilot trial. The study assessed cue‐induced craving using a visual analogue scale before and after administering 600 mg CBD once daily for 3 consecutive days. The findings indicated a significant reduction in cue‐induced craving following CBD dosing. The study did not find any significant changes in scores for depression, anxiety, pain or opioid withdrawal symptoms.

In another double‐blind placebo‐controlled cross‐over pilot trial, Suzuki et al. [40] investigated the impact of CBD on reward‐ and stress‐related neurocognitive processes among individuals with OUD receiving treatment with buprenorphine or methadone. In this cross‐over study, participants either received a single dose of CBD (600 mg) or a placebo during each of the two test sessions, and cue‐induced craving (measured by a visual analogue scale) and attentional bias toward drug‐related cues (measured by a visual probe task) were assessed. Additional assessments, including decision‐making, delayed discounting, distress tolerance, stress reactivity, opioid withdrawal, mood states and vital signs, were also explored. Findings revealed that a single dose of CBD significantly reduced cue‐induced craving and attentional bias toward drug‐related cues, while other measures remained unchanged.

#### 3.1.2 Safety Profile

The safety profile of CBD in combination with opioids was first assessed in a double‐blind, placebo‐controlled, cross‐over study by Manini et al. [41]. In this study, the safety and pharmacokinetics of orally administered CBD in combination with intravenous fentanyl were investigated in healthy participants with prior opioid exposure. The results of the study showed that CBD at doses of 400 and 800 mg did not exacerbate the adverse effects associated with fentanyl, and the coadministration of CBD and fentanyl was safe and well‐tolerated.

#### 3.1.3 Quality Assessment

**FIGURE 2:**
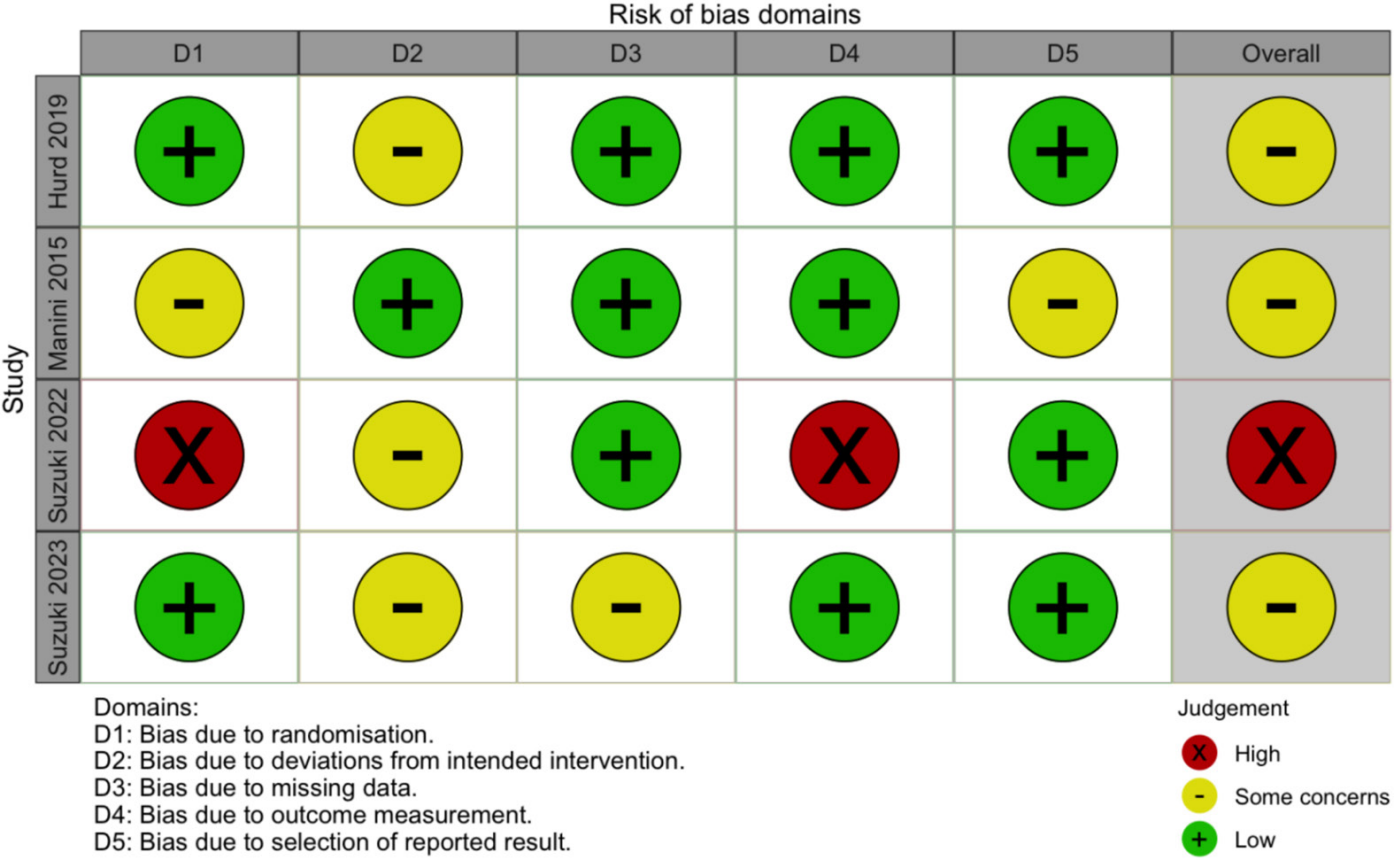
Risk of bias assessments for clinical studies using the revised Cochrane risk‐of‐bias tool for randomized trials (RoB 2). Suzuki et al.'s (2022) study was a single‐arm open‐label pilot study.

**FIGURE 3:**
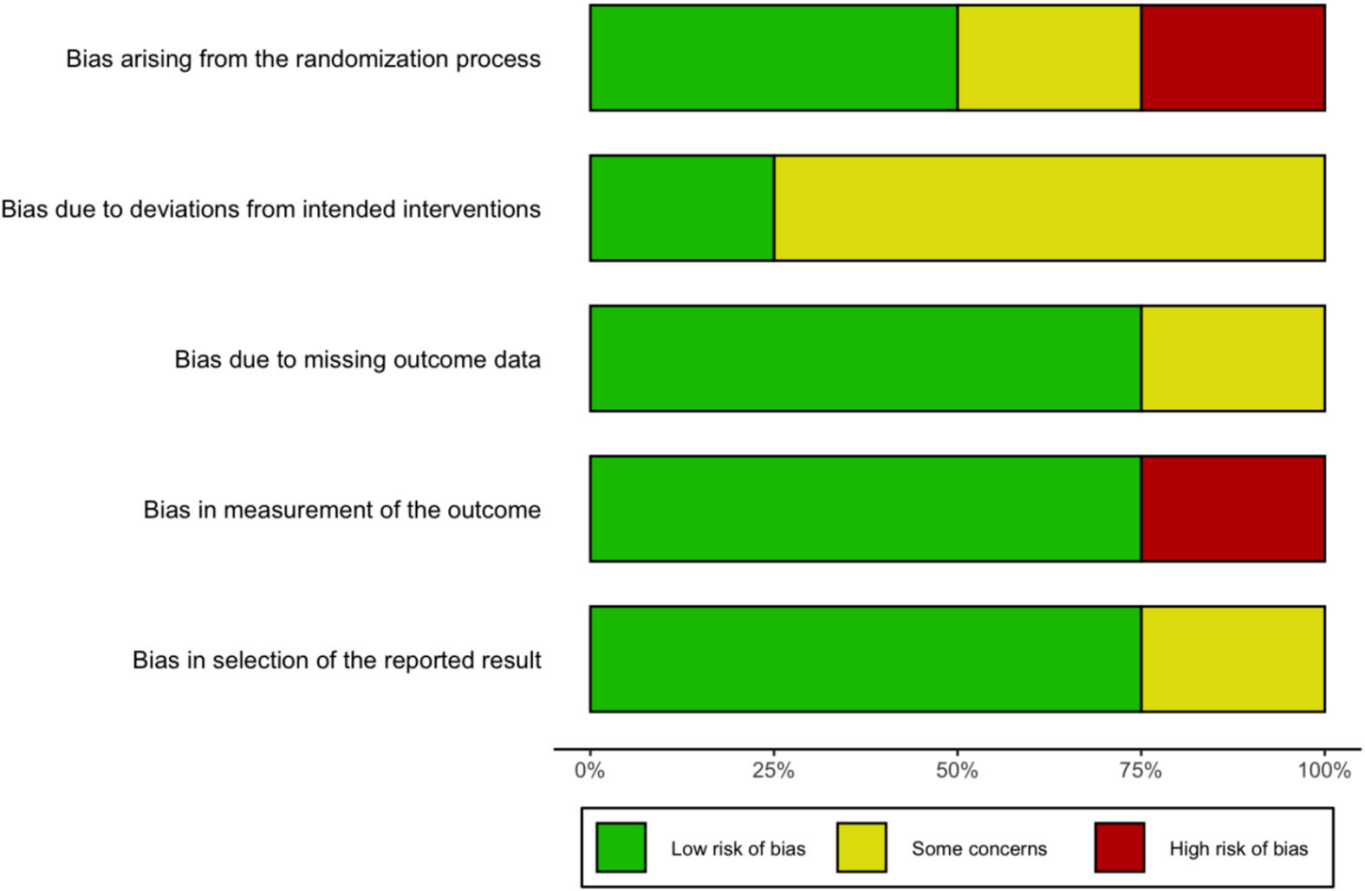
Summary of risk of bias assessments for clinical studies.

The ROB assessment using the Cochrane ROB 2 tool for the clinical studies is presented in Figures 2 and 3. Three studies, Manini et al. [41], Hurd et al. [38] and Suzuki et al. (2023) [40], exhibited an overall assessment of ‘some concerns’. It is important to note that Suzuki et al. [39] was a pilot open‐label study, which was not expected to fully satisfy the ROB tool criteria for randomized clinical trials.

### 3.2 Preclinical Studies

This systematic review identified 16 preclinical studies that investigated the use of CBD in opioid‐induced animal models. The studies were published between 1975 and 2023, and there were seven mouse models, eight rat models and one primate model experimental study (Table 2). Here, we have systematically categorized the literature based on the primary models utilized in each study. Some studies employed multiple models to evaluate the effects of CBD.

**TABLE 2:** Characteristics of reviewed preclinical studies.

No.	Study/country	Objective	Experimental approach	Subjects	Sample size (*N*)	Opioid model	Cannabinoids	Frequency and duration of treatment in each arm	Outcome results
**Opioid withdrawal models**									
1	Hine and Friedman et al. (1975)/United States	Effects of THC and CBD on morphine‐dependent rats with naloxone precipitated morphine abstinence	Experimental rat model—Abstinence signs	Adolescent male rats (170–190 g)	35	Morphine SC single implantation containing 75 mg	CBD and THC	THC (1, 2, 5 or 10 mg/kg) or CBD (10 mg/kg) IP	THC 5 and 10 mg/kg significantly reduced the frequency of wet shakes and escapes and reduced abstinence scores (*p* < 0.05). CBD alone did not reduce abstinence.
2	Hine and Torrelio et al. (1975)/United States	Effect of CBD, THC and CBD + THC on morphine‐dependent rats with naloxone precipitated morphine abstinence	Experimental rat model—Abstinence signs	Adolescent male rats (170–190 g)	33	Morphine SC single implantation containing 75 mg	CBD and THC	CBD (10 mg/kg) followed by THC (2 mg/kg) IP	CBD did not reduce the morphine abstinence score. CBD reduced morphine abstinence when administered with THC. A synergistic effect was noted with CBD + THC more effective at reducing abstinence scores than THC alone (*p* < 0.05).
3	Bhargava (1976)/United States	Effects of various cannabinoids on naloxone‐precipitated withdrawal in morphine‐dependent mice in suppressing morphine abstinence signs	Experimental mouse model—Dose of naloxone needed to induce withdrawal jumping in 50% of the animals (ED50)	Male Swiss‐Webster mice (25–30 g)	NR	Morphine SC single implantation containing 75 mg	THC, delta8‐THC, 11‐hydroxy‐delta8‐THC, CBD, CBN	Each (5 and 10 mg/kg) IP once	All of the cannabinoids inhibited the naloxone‐precipitated morphine abstinence (increase in the naloxone ED50). Effectiveness: THC > delta8‐THC > 11‐hydroxy‐delta8‐THC>CBD > CBN.
4	Chesher and Jackson (1985)/Australia	Effect of CBN, CBD and THC on QMWS	Experimental rat model—QMWS protocol	Male Sprague–Dawley rats	200	IBMX 15 mg/kg single SC	CBD, CBN and THC	CBD (5, 20 or 80 mg/kg), CBN (5, 20 or 80 mg/kg) and THC (5 and 10 mg/kg) IP	CBD did not decrease the mean withdrawal score.
5	Scicluna (2022)/Australia	Effect of CBD on reducing the severity of gastrointestinal symptoms during opioid withdrawal in male and female mice	Experimental mouse model—Withdrawal symptoms	Male and female C57BL/6J mice aged 9–10 weeks (17–26 g)	268	Oxycodone hydrochloride (9, 17.8, 23.7 and 33 mg/kg) IP twice daily on Days 1–2, 3–4, 5–6 and 7–8 and a single 33 mg/kg dose on Day 9	CBD	CBD (10, 30 or 100 mg/kg) IP 1 h before withdrawal testing	CBD dose‐dependently reduced gastrointestinal symptoms during both PW and SW in male mice and during PW in female mice. CBD had no effect on PW‐ or SW‐induced jumping in male mice, but in female mice, the PW‐induced increase in jumps was less pronounced in CBD‐treated mice. The highest dose of CBD inhibited paw tremors during PW in male mice but not during SW. Neither PW‐ nor SW‐induced paw tremors were observed in female mice.
**Conditioned place preference and aversion models**									
6	de Carvalho and Takahashi (2017)/Brazil	Effect of CBD on reconsolidation of contextual drug‐associated memories in rats using morphine and cocaine CPP paradigm and naltrexone conditioned place aversion	Experimental rat model—CPP paradigm	Young adult Wistar male rats (120–180 g)	295	Morphine, cocaine 2.5 and 10 mg/kg SC	CBD	CBD (5 or 10 mg/kg) SC	CBD disrupted the reconsolidation of preference for the environments induced by morphine and cocaine. Preference was not restored after further reinstatement induced by priming drug or stress reinstatement. CBD significantly reduced morphine‐CPP and suppressed subsequent naltrexone‐precipitated CPA.
7	Markos et al. (2018)/United States	Effect of CBD on the development of morphine morphine‐conditioned Place preference in mice	Experimental mouse model—CPP paradigm	Adult male mice (25–30 g)	100	Morphine 2.5 mg/kg/mL IP	CBD	CBD solutions (2.5, 5, 10 and 20 mg/kg/mL) IP	CBD 10 mg/kg group significantly decreased preference scores compared to the vehicle group (*p* = 0.033). CBD had no rewarding and aversive properties.
8	Harris et al. (2022)/United States	Effects of CBD and a novel CBD analogue CBD‐val‐HS on oxycodone place preference and analgesia in mice	Experimental mouse model with the control group—CPP paradigm	C57BL/6 male mice (25–30 g)	NR	Oxycodone (Tocris, Boston, MA) in 0.9% saline at 3 mg/mL	CBD and CBD‐val‐HS	Each (1.0 mL/kg) IP	CBD did not attenuate oxycodone place preference, while CBD‐val‐HS attenuated these rewarding effects at 8.0 mg/kg and was void of rewarding or aversive properties. CBD‐val‐HS produced an analgesic effect compared to oxycodone in nociceptive assays, especially thermal nociception.
9	Souza et al. (2023)/Brazil	Effect of CBD on the expression of CPA induced by naloxone‐precipitated morphine withdrawal by the involvement of 5‐HT1A receptors	Experimental mouse model—CPA paradigm	Male C57BL/6 mice aged 6–8 weeks	NR	Morphine Day 1: 10 mg/kg; Day 2: 30 mg/kg; Day 3: 50 mg/kg (twice a day) and Day 4: 60 mg/kg (only one injection in the morning) IP	CBD	CBD (15, 30 and 60 mg/kg) IP 30 min before the CPA	CBD 30 and 60 mg/kg attenuated the expression of conditioned place aversion, possibly through the activation of 5‐HT1A receptors.
**Self‐administration models**									
10	Ren et al. (2009)/United States	Effect of CBD on cue‐induced heroin self‐administration and drug‐seeking behaviour	Experimental rat model—Heroin self‐administration and drug‐seeking behaviour after drug reinstatement or light conditioned cue	Young adult male rats (230–250 g)	155	Heroin (30 μg/kg/infusion)	CBD	CBD (5 or 20 mg/kg) IP	CBD did not modify stable heroin self‐administration CBD attenuated heroin‐seeking behaviours (*p* < 0.05) for 2 weeks.
**Multiple approaches**									
11	Hudson et al. (2019)/Canada	Opposite effects of intra‐vHipp THC and CBD on opioid reward processing via local ERK1–2 modulation	Experimental rat model—In vivo electrophysiology, CPP and fear conditioning assays, ERK1–2 signalling	Male Sprague–Dawley rats (250–300 g)	NR	Morphine 0.05 mg/kg IP	THC (Cayman Chemical) and CBD (Tocris Bioscience)	Intra‐vHipp microinfusions were performed immediately before each behavioural assay or conditioning session	THC significantly increased the percentage of time spent in the morphine context relative to VEH (*p* < 0.035), CBD (*p* < 0.028) and THC + CBD (*p* < 0.007) groups. Receiving THC + CBD coadministration demonstrated a greater percentage of time spent in the saline versus morphine‐paired contexts (*p* < 0.039). Rats receiving THC + U0126 (MEK1–2 inhibitor) did not differ from VEH in percentage time spent in the morphine context. Relative to rats receiving VEH (*p* < 0.022), those receiving THC + CBD + EPA increased the percentage of time spent in the morphine context. CBD coadministration reverses the potentiation of reward memory salience induced by intra‐vHipp THC via local pERK1–2 inhibition.
12	Navarrete et al. (2022)/Spain	Effect of CBD on the behavioural and gene expression alterations induced by spontaneous heroin withdrawal	Experimental mouse model—Withdrawal‐related behaviour and gene expression changes in specific brain regions	CD1 male mice	90	Heroin starting with 5 mg/kg/12 h (SC) at Day 1 and rising to 40 mg/kg/12 h (SC) at Day 8	CBD	CBD (5, 10 and 20 mg/kg) IP	CBD significantly reduced behavioural impairments and normalized gene expression of Cnr1 and Pomc in the NAcc and TH in the VTA of mice exposed to spontaneous heroin withdrawal. CBD induced an upregulation of Cnr2, whereas it did not change the increased gene expression of Oprm1 in the NAcc of abstinent animals.
13	Jin et al. (2023)/China	Effect of the CBD derivative CIAC001 in treating morphine‐induced addiction by targeting PKM2	Experimental mouse model, including in vitro inflammatory model and in vivo withdrawal symptoms and CPP paradigm	BALB/c male mice, aged 6–8 weeks (20–28 g)	NR	Morphine IP three times daily for 3 days (5, 20 and 40 mg/kg). On the fourth day, mice were given morphine 40 mg/kg.	CIAC001, CBD	The frequency and duration of treatment in each arm varied depending on the specific experiment	In vitro CIAC001 exhibited significantly improved antineuroinflammatory activity with lower toxicity. In vivo CIAC001 ameliorated the morphine‐induced withdrawal reaction, behavioural sensitization and conditional position preference by inhibiting morphine‐induced microglia activation and neuroinflammation. Target fishing for CIAC001 by activity‐based protein profiling led to the identification of pyruvate kinase M2 as the target protein.
14	Rivera‐Garcia (2023)/United States	Effect of inhaled high‐CBD WPE on enhancing the antinociceptive effects of opioids, reducing opioid tolerance and attenuating opioid reward in female rats	Experimental rat model, CPP paradigm and fentanyl self‐administration	Young adult female Long Evans rat	196	Morphine (10 mg/kg) SC twice daily	WPE with a composition of 64.2% CBD and 7.1% THC	Chronic exposure to WPE: Twice daily for 20 days Single session exposure to WPE: 30‐min session with 15 5‐s vapour deliveries	Chronic exposure to high‐CBD WPE did not have adverse effects on lung cytoarchitecture, estrous cycle, cognitive function, social behaviour or anxiety levels. WPE inhalation prevented morphine‐induced conditioned place preference and reinstatement and reduced fentanyl self‐administration in rats with and without neuropathic pain. High‐CBD vapour has modest analgesic effects, a robust safety profile, no abuse potential and significantly reduces opioid reward.
**Other models**									
15	Katsidoni et al. (2013)/Greece	Effect of CBD on brain stimulation reward and on morphine‐ and cocaine‐ reward facilitating effect	Experimental rat model, ICSS paradigm	Young adult male rats (300–350 g)	NR	Morphine 1 mg/kg SC, cocaine 1 mg/kg IP	CBD	CBD (5, 10 or 20 mg/kg) IP For intracranial injections, guide cannula into the dorsal raphe	CBD at 10 and 20 mg/kg doses increased the ICSS threshold (*p* < 0.001). CBD inhibited the reward‐facilitating effect of morphine (but not cocaine). CBD inhibited the decreased ICSS threshold effect of morphine. 5‐HT1A antagonist reversed the impact of CBD on the reward‐facilitating effect of morphine.
16	Carey et al. (2023)/United States	Effects of delta‐THC, CBD and THC/CBD mixtures on reinforcing effects of fentanyl versus food choice paradigm in rhesus monkeys	Experimental animal model, food versus drug choice paradigm	Adult male rhesus monkeys	4	Fentanyl hydrochloride (0.0001 mg/kg for all four subjects, 0.001 mg/kg for two subjects and 0.0032 mg/kg for other two)	THC, CBD and THC + CBD (1:10 and 1:32)	Various doses IV	CBD did not alter the choice for large dose fentanyl (*p* = 0.66) or small dose (*p* = 0.32). Differences were observed in one monkey, with the effects of CBD at 10 mg/kg decreasing large dose fentanyl choice by 40%.

Abbreviations: CBD: cannabidiol, CBN: cannabinol, CPA: conditioned place aversion, CPP: conditioned place preference, IBMX: 3‐isobutyl‐l‐methylxanthine, ICSS: intracranial self‐stimulation, IP: intraperitoneal, IV: intravenous, NA: not applicable, NR: not reported, PW and SW: naloxone‐precipitated and spontaneous withdrawal, QMWS: quasimorphine withdrawal syndrome, SC: subcutaneous, THC: tetrahydrocannabinol, WPE: whole‐plant cannabis extract.

#### 3.2.1 Opioid Withdrawal Models

While a few studies showed significant effects of CBD on reducing opioid withdrawal symptoms [42, 43], some others did not find any effects or only reported its effectiveness in combination with THC [44, 45, 46].

Bhargava et al. [42] investigated the effects of various intraperitoneal cannabinoids on naloxone‐precipitated withdrawal in morphine‐dependent mice. The study found that all the cannabinoids inhibited the naloxone‐precipitated opioid withdrawal syndrome, as evidenced by an increase in the naloxone ED50 and suppression of opioid withdrawal symptoms, such as defecation and rearing behaviour. The relative effectiveness of the cannabinoids in inhibiting opioid withdrawal syndrome appeared to follow the order of delta9‐THC > delta8‐THC > 11‐hydroxy‐delta8‐THC > CBD > cannabinol (CBN). Scicluna et al. [43] investigated the efficacy of CBD on reducing the severity of gastrointestinal symptoms during opioid withdrawal in male and female mice and reported dose‐dependent effects of CBD on the reduction of the gastrointestinal symptoms during both precipitated and spontaneous withdrawal in male mice. Additionally, CBD inhibited precipitated withdrawal‐induced paw tremors in males and jumps in female mice.

However, two other early studies did not find any significant effects of CBD on opioid withdrawal symptoms. Hine and Friedman et al. [45] aimed to determine the effect of THC and CBD on naloxone‐precipitated morphine withdrawal symptoms in morphine‐dependent rats. While THC at higher doses significantly reduced the frequency of wet shakes, escapes and abstinence scores, CBD did not reduce any of the opioid withdrawal symptoms. In the next step, Hine and Torrelio et al. [46] investigated the interaction of CBD and THC on morphine abstinence with a similar design. Similar to the previous study, CBD alone did not significantly reduce morphine withdrawal symptoms, while THC significantly did. However, there was a synergistic effect observed with the combination of CBD and THC, indicating greater efficacy in reducing withdrawal symptoms. Similarly, Chesher and Jackson [44] investigated the effect of different cannabinoids, including CBN, CBD and THC, on quasimorphine withdrawal syndrome (QMWS) in rats in a placebo‐controlled experiment. Compared to placebo, none of the three doses of CBD (5, 20 or 80 mg/kg) could decrease the mean withdrawal score, whereas THC and CBN significantly lowered it.

#### 3.2.2 CPP and Aversion Models

A few animal models used the CPP paradigm to investigate the effects of CBD on the opioid administration rewarding effects or opioid + naltrexone aversion model. These studies demonstrated that CBD attenuates opioid CPP and opioid + naltrexone aversive effects. de Carvalho and Takahashi [47] examined the effects of CBD on the reconsolidation of contextual drug‐associated memories in rats using a CPP paradigm with morphine, cocaine and naltrexone‐conditioned place aversion. This study found that CBD significantly reduced morphine‐CPP and suppressed subsequent naltrexone‐precipitated conditioned place aversion. Similarly, Markos et al. [48] evaluated the effects of various doses of CBD on the development of morphine‐CPP in mice and found that CBD at a dose of 10.0 mg/kg significantly lowered preference scores compared to the vehicle group.

The potential differences between CBD and a novel CBD analogue, CBD‐val‐HS, were investigated by Harris et al. [49] who reported that CBD did not attenuate oxycodone place preference while CBD‐val‐HS decreased CPP effects at a dose of 8 mg/kg. Additionally, CBD‐val‐HS alone produced an analgesic effect, specifically in a nociceptive hot plate assay. In an experimental mouse model, Souza et al. [50] explored the effects of CBD on the expression of conditioned place aversion induced by naloxone‐precipitated morphine withdrawal. The results demonstrated that CBD administration at doses of 30 and 60 mg/kg attenuated the expression of conditioned place aversion.

#### 3.2.3 Self‐Administration Models

We found one study on the effects of CBD on cue‐induced heroin self‐administration and drug‐seeking behaviours. In an experimental rat model, Ren et al. [51] reported that CBD did not modify heroin self‐administration; however, it could specifically reduce drug‐induced reinstatement and attenuate heroin‐seeking behaviours.

#### 3.2.4 Multiple Approaches

As a part of a study investigating psychotropic side effects of intra‐vHipp THC and CBD, Hudson et al. [52] explored the effects of intra‐vHipp THC and CBD on opioid reward processing in male rats. THC significantly increased the percentage of time spent in the morphine context relative to the vehicle, whereas CBD reduced the time spent in the morphine context. Moreover, CBD coadministration with THC reversed the potentiation of reward memory salience induced by THC.

Navarrete et al. [53] investigated the effects of CBD on behavioural and gene responses in heroin‐exposed male mice. The study utilized a mouse model and found that CBD at doses of 5, 10 and 20 mg/kg significantly reduced behavioural responses associated with heroin withdrawal, such as anxiety‐like behaviour, motor activity and somatic signs. Additionally, CBD normalized the associated gene expression changes such as MOR, proopiomelanocortin, cannabinoid receptors and tyrosine hydroxylase.

Jin et al. [54] reported that CIAC001, a CBD derivative, significantly improved antineuroinflammatory activity in vitro and ameliorated the morphine‐induced withdrawal reaction, behavioural sensitization and CPP in vivo by inhibiting morphine‐induced microglia activation and neuroinflammation.

The effects of inhaled high‐CBD whole‐plant cannabis extract (WPE) on antinociceptive effects of opioids, opioid tolerance and opioid reward were investigated in female rats by Rivera‐Garcia et al. [55]. The experimental rat model involved chronic exposure to WPE through vapour inhalation. The findings demonstrated that chronic exposure to high‐CBD WPE prevented morphine‐induced CPP and reinstatement and reduced fentanyl self‐administration in rats with and without neuropathic pain. Moreover, high‐CBD WPE did not have adverse effects on lung cytoarchitecture, estrous cycle, cognitive function, social behaviour or anxiety levels.

#### 3.2.5 Other Models

Katsidoni et al. [56] examined the effect of CBD on brain stimulation reward and morphine‐ and cocaine‐reward facilitating effects in rats using an intracranial self‐stimulation (ICSS) paradigm. CBD at 10 and 20 mg/kg doses increased the ICSS threshold, indicating a decrease in the rewarding effects of morphine. In addition, CBD inhibited the reward‐facilitating effect of morphine but not cocaine. Another study by Carey et al. [57] examined the effects of delta‐THC, CBD and THC/CBD mixtures on the reinforcing effects of fentanyl in a drug versus food choice paradigm in rhesus monkeys. The results of this study failed to show any effect of CBD on the choice of any doses of fentanyl. However, individual differences were highlighted in one monkey for which 10 mg/kg CBD decreased preference for a high dose of fentanyl by 40%.

#### 3.2.6 Quality Assessment

**FIGURE 4:**
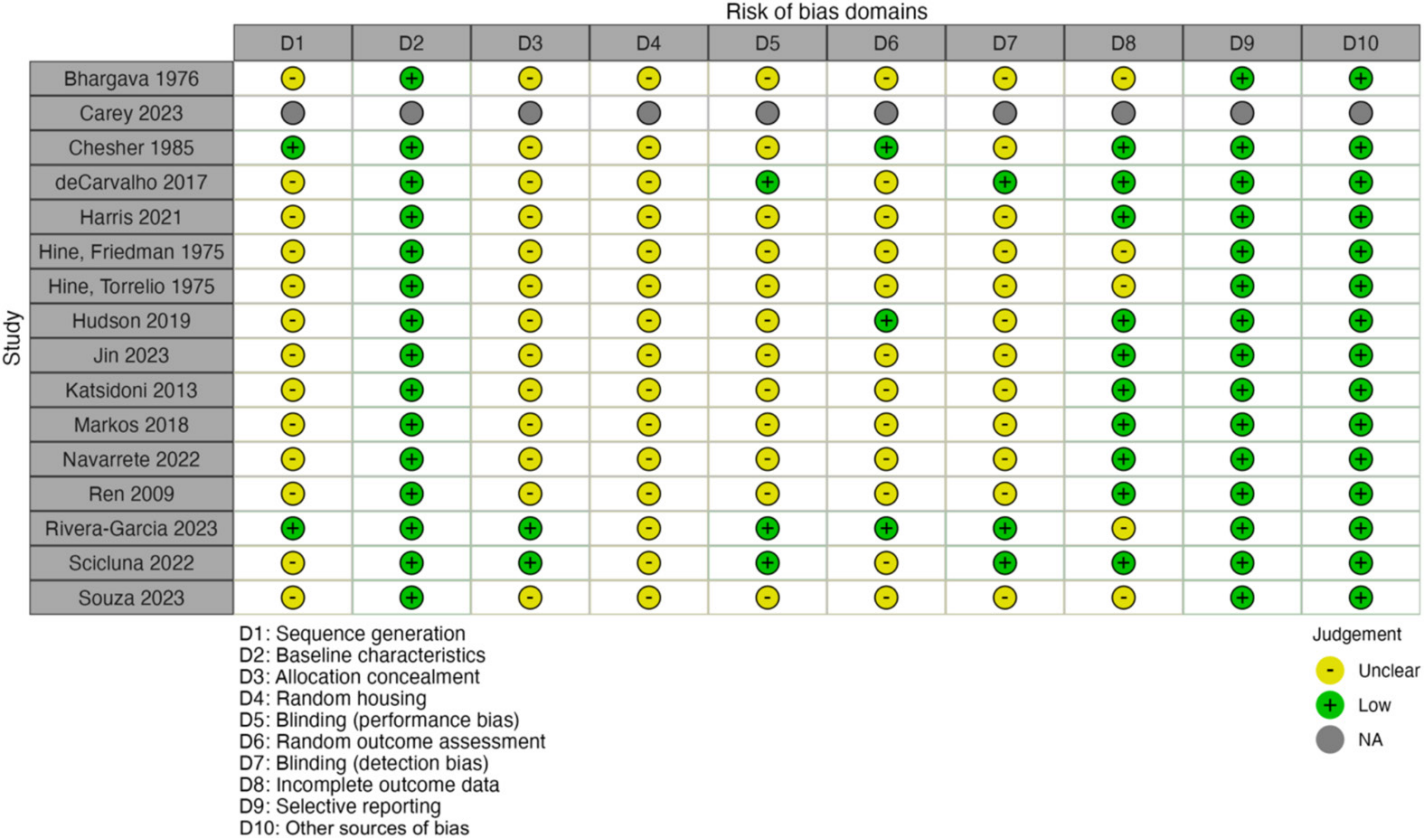
Risk of bias assessments for animal studies using the SYRCLE's risk of bias tool. Carey et al.'s (2023) study examined the effects of THC and CBD in four rhesus monkeys and was not a trial.

**FIGURE 5:**
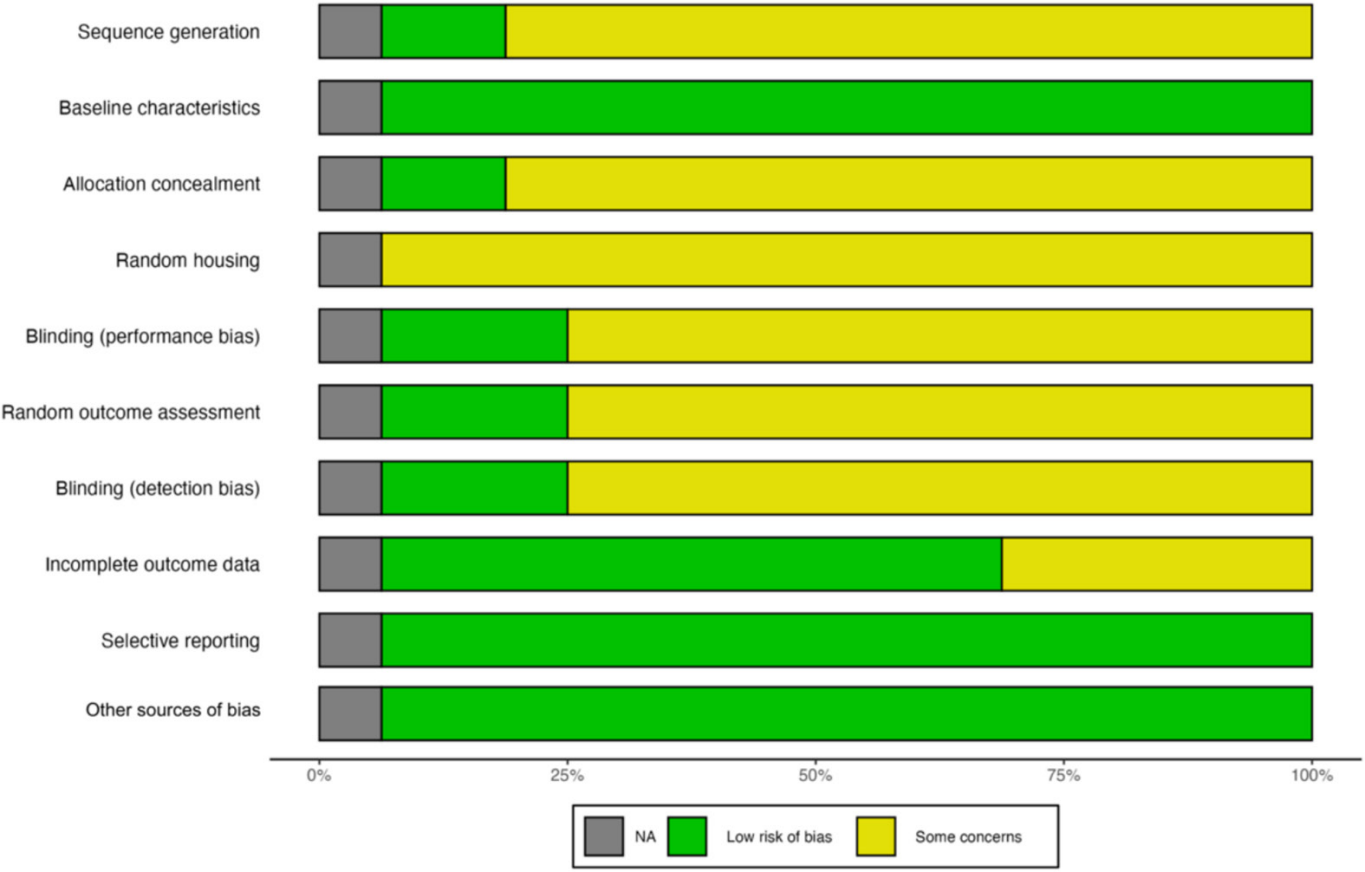
Summary of risk of bias assessments for animal studies.

The results of the ROB assessment for preclinical studies, as detailed in Figures 4 and 5, indicated a considerable degree of ‘unclear’ risk across the evaluated domains. Notably, the study by Carey et al. [57] was performed on four rhesus monkeys and provided no applicable data on the ROB across all domains. Conversely, Rivera‐Garcia et al. [55] and Scicluna et al. [43] presented more balanced assessments, with ‘low risk’ ratings across most domains. Overall, the preclinical studies generally tended toward ‘unclear risk’ in most domains, highlighting the lack of reporting and methodological transparency in these animal studies.

## 4 Discussion

In this systematic review, we presented an overview of the current evidence on CBD's potential therapeutic effects and safety profile in clinical and preclinical studies of OUD. The overall findings support that CBD is well‐tolerated during opioid use and withdrawal, and its administration is associated with reductions in opioid craving and anxiety, although the results concerning its effectiveness in alleviating opioid withdrawal symptoms and reducing opioid rewarding effects are mixed.

The reviewed clinical studies align with previous safety research on CBD [58] and indicate that CBD is well‐tolerated and safe for human administration. Specifically, CBD was shown to be well‐tolerated at doses up to 800 mg and did not enhance the effects of opioids, with no reports of respiratory depression or cardiovascular complications during the trials. Additionally, when coadministered with opioids, the pharmacokinetics and clearance of CBD remained unchanged. This is particularly important when considering CBD as a therapeutic intervention for OUD. While the clinical trials reviewed did not report any major adverse events, other trials involving different patient populations reported an elevation of liver enzyme following CBD administration and, in rare cases, CBD‐induced liver injury, especially at daily doses exceeding 1000 mg or when used with other antiepileptic medications. Notably, no cases of liver injury have been reported in adults using CBD doses below 300 mg/day, and no instances of severe drug‐induced liver injury (DILI) were documented [59].

Clinical studies investigating the potential therapeutic effects of CBD in OUD treatment have primarily focused on mitigating opioid cravings and anxiety, as these factors could significantly reduce the risk of relapse in individuals with OUD. The available clinical studies have demonstrated that CBD significantly reduces anxiety and cue‐induced cravings in individuals with OUD [38, 39, 40]. The anxiolytic properties of CBD have also been documented in other psychiatric disorders, including individuals with generalized anxiety disorder (GAD), social anxiety and the anxiety component of post‐traumatic stress disorder (PTSD) [60]. Moreover, CBD has been shown to lower cortisol levels and diminish autonomic arousal and physiological measures of stress reactivity [61, 62]. Neuroimaging studies indicate that these anxiolytic effects are associated with the modulation of limbic and paralimbic structures, as CBD reduces activity in these neural circuits during negative emotional processing [63]. While not reported by all studies, some studies demonstrated that CBD administration alleviates negative affective scores [39, 40]. Nevertheless, the amelioration of features central to substance use disorders, such as craving, may be closely related to CBD's effects on emotional processing, stress regulation and anxiety [61, 62].

As an emerging therapeutic option for OUD, future research is essential to address several important questions. While current evidence supports the beneficial effects of a short course of CBD in reducing opioid craving, the long‐term effects of CBD on these symptoms are unknown. Moreover, it has yet to be determined whether CBD‐induced reduction in opioid craving will lead to a decreased risk of relapse or illicit opioid use in individuals with OUD. It is also crucial to investigate whether CBD is more effective as an adjunct medication or if it can be used as a standalone treatment. In the reviewed trials, CBD was used independently in one study [38] and as an adjunct to buprenorphine [39] and buprenorphine or methadone [40] in two others, primarily focusing on the abstinence and early recovery stages. This underscores the necessity for further research to identify the specific stages of OUD at which CBD is most effective, both with and without existing OUD medications [64].

One of the main focuses of preclinical studies has been the effects of CBD on opioid withdrawal symptoms [44, 45, 46]. Specifically, research has demonstrated that CBD can reduce several signs of opioid withdrawal, such as defecation, tremor, rearing, rubbing, grooming, jumping and digging. These effects appear to be mediated through interactions with the opioidergic, dopaminergic and cannabinoid systems [65]. It has also been proposed that CBD may normalize gene expression changes associated with opioid withdrawal, particularly in the NAc [53]. Some studies also demonstrated that CBD ameliorates anxiogenic responses and somatic withdrawal signs in animal models of opioid withdrawal [42, 43].

Other preclinical studies investigated the rewarding effects of opioids using the CPP paradigm. These studies have demonstrated that CBD can attenuate the rewarding effects of opioids [47, 48, 50], although not all studies have found similar results [49]. It has been suggested that variations in the rewarding properties of various opioids may influence the effect of CBD and its effective doses. Notably, CBD may not block the reward associated with oxycodone, possibly due to oxycodone's greater analgesic and rewarding effects compared to morphine, which has been studied more frequently [66, 67]. Importantly, unlike THC, CBD does not impair learning or memory, indicating that its effects in the CPP paradigm are not related to cognitive disturbances [68]. This body of evidence suggests that the eCB system may play a significant role in mediating the opioid reward pathway. The CBD's effects on reducing opioid reward could have significant clinical implications in relapse prevention, addressing a major challenge in OUD treatment.

The eCB system closely interacts with various neurotransmission systems, significantly influencing the neural adaptations associated with addiction. Research on various cannabinoids has shown that these compounds exert distinct neurobiological effects and target different pathways. While it is well established that THC acts as a partial agonist at cannabinoid receptors [69], the precise molecular mechanism of action of CBD warrants further investigation in future studies [29]. Some reports indicate that CBD functions as an inverse agonist at both CB1R and CB2R [70], while others suggest that it may indirectly enhance endogenous AEA signalling by inhibiting its intracellular degradation, which is catalysed by the enzyme FAAH [71]. Moreover, CBD allosterically modulates μ‐ and δ‐opioid receptors [72]. CB1 receptors and μ‐opioid receptors are locally associated and share Gi‐alpha‐mediated intracellular signalling in several brain regions, including NAc and dorsal striatum [73]. This interaction has implications for reward processing, goal‐directed behaviour and habit formation, all of which are relevant to addiction [74]. Additionally, other mechanisms attributed to CBD include its action as an allosteric agonist of the serotonin 5‐HT1A receptor [75], a weak inhibitor of dopamine uptake in the striatum [76] and interactions with glutamate‐GABA pathways [77]. Recent studies suggest that the antineuroinflammatory activity of CBD derivatives, particularly through the inhibition of microglia in the mPFC, plays a significant role in blocking morphine‐induced withdrawal reactions, behavioural sensitization and CPP [54]. Further studies are necessary to deepen our understanding of how CBD affects OUD and its underlying neurobiological mechanisms.

### 4.1 Limitations

Although conducting a comprehensive search using a broad strategy to include all clinical and preclinical studies on the use of CBD in OUD, only four clinical trials, including two pilot studies, were identified, limiting the generalizability of the findings. While the studies investigated a diverse population regarding sex, race and ethnicity, the small trial sizes were a major limitation. Additionally, several participants had psychiatric comorbidities, which, although reflective of real‐world conditions, may have influenced the outcomes. Moreover, the follow‐up assessments were typically conducted approximately 1 week after the final CBD dose, indicating a short follow‐up period for assessing efficacy and safety. Furthermore, the heterogeneity in study design, experimental approaches, participant characteristics and findings presents significant challenges in drawing clear conclusions from preclinical studies, highlighting the need for consistent protocols and standardized outcome measures in future research.

Despite the encouraging findings, large‐scale, randomized controlled trials with long‐term follow‐up are essential to confirm the efficacy and safety of CBD, elucidate its role in comprehensive addiction treatment programs, identify the stage of OUD treatment where CBD is most effective and determine the optimal dosing and long‐term effects of CBD in OUD treatment. Additionally, exploring the synergistic effects of CBD in combination with current pharmacotherapies available for OUD could provide valuable insights into integrated treatment approaches for OUD.

## 5 Conclusion

This systematic review highlights the potential of CBD as a beneficial treatment option for OUD. The collective evidence from clinical and preclinical studies indicates that CBD holds significant promise as a novel therapeutic option for OUD treatment with an acceptable safety profile. CBD's effects on the reduction of opioid cravings (as shown in clinical studies) and its potential to diminish the rewarding effects of opioids and alleviate withdrawal symptoms (as indicated by some preclinical studies) could present a significant advancement in OUD treatment. Continued research will be crucial in confirming these findings and establishing CBD as a valuable component of OUD treatment.

## Author Contributions

All authors contributed to developing the selection criteria, risk of bias assessment strategy and data extraction criteria. All authors read, provided feedback and approved the final manuscript.

## Ethics Statement

The authors have nothing to report.

## Consent

The authors have nothing to report.

## Conflicts of Interest

Dr. Bassir Nia is a member of the Scientific Advisory Board of Synendos Therapeutics AG, Switzerland. The other authors declare no conflicts of interest.

## Supporting information


**Table S1.** The search strategy of databases.

## Data Availability

Data sharing is not applicable to this article as no new data were created or analyzed in this study.

## References

[1] J. Shen , G. Hua , C. Li , S. Liu , L. Liu , and J. Jiao , “Prevalence, Incidence, Deaths, and Disability‐Adjusted Life‐Years of Drug Use Disorders for 204 Countries and Territories During the Past 30 Years,” Asian Journal of Psychiatry 86 (2023): 103677. 37348194 10.1016/j.ajp.2023.103677

[2] C. Florence , F. Luo , and K. Rice , “The Economic Burden of Opioid Use Disorder and Fatal Opioid Overdose in the United States, 2017,” Drug and Alcohol Dependence 218 (2021): 108350. 33121867 10.1016/j.drugalcdep.2020.108350 PMC8091480

[3] J. J. Carroll , T. C. Green , and R. K. Noonan , Evidence‐Based Strategies for Preventing Opioid Overdose: What's Working in the United States: An Introduction for Public Heath, Law Enforcement, Local Organizations, and Others Striving to Serve Their Community, (2018).

[4] T. Mariolis , J. Bosse , S. Martin , A. Wilson , and L. Chiodo , “A Systematic Review of the Effectiveness of Buprenorphine for Opioid Use Disorder Compared to Other Treatments: Implications for Research and Practice,” Journal of Addiction Research & Therapy 10, no. 2 (2019): 1000379.

[5] D. Dowell , “Treatment for Opioid Use Disorder: Population Estimates—United States, 2022,” MMWR. Morbidity and Mortality Weekly Report 73, no. 25 (2024): 567–574, 10.15585/mmwr.mm7325a1. 38935567 PMC11254342

[6] N. C. Cardamone , R. E. Stewart , K. M. Kampman , and S. C. Marcus , “Perspectives of Substance Use Disorder Counselors on the Benefits and Drawbacks of Medications for Opioid Use Disorder,” Addiction Science & Clinical Practice 20, no. 1 (2025): 7. 39905512 10.1186/s13722-025-00537-2 PMC11792642

[7] A. M. Aghaei , A. Saali , M. A. Canas , et al., “Dysregulation of the Endogenous Cannabinoid System Following Opioid Exposure,” Psychiatry Research 330 (2023): 115586, 10.1016/j.psychres.2023.115586. 37931479 PMC10842415

[8] J. L. Scavone , K. Mackie , and E. J. Van Bockstaele , “Characterization of Cannabinoid‐1 Receptors in the Locus Coeruleus: Relationship With mu‐Opioid Receptors,” Brain Research 1312 (2010): 18–31. 19931229 10.1016/j.brainres.2009.11.023 PMC2835571

[9] J. A. Lopez‐Moreno , A. Lopez‐Jimenez , M. A. Gorriti , and F. R. de Fonseca , “Functional Interactions Between Endogenous Cannabinoid and Opioid Systems: Focus on Alcohol, Genetics and Drug‐Addicted Behaviors,” Current Drug Targets 11, no. 4 (2010): 406–428. 20196742 10.2174/138945010790980312

[10] J. M. Wenzel and J. F. Cheer , “Endocannabinoid Regulation of Reward and Reinforcement Through Interaction With Dopamine and Endogenous Opioid Signaling,” Neuropsychopharmacology 43, no. 1 (2018): 103–115. 28653666 10.1038/npp.2017.126 PMC5719091

[11] A. N. M. Schoffelmeer , F. Hogenboom , G. Wardeh , and T. J. De Vries , “Interactions Between CB1 Cannabinoid and μ Opioid Receptors Mediating Inhibition of Neurotransmitter Release in Rat Nucleus Accumbens Core,” Neuropharmacology 51, no. 4 (2006): 773–781. 16806307 10.1016/j.neuropharm.2006.05.019

[12] D. L. Cichewicz , “Synergistic Interactions Between Cannabinoid and Opioid Analgesics,” Life Sciences 74, no. 11 (2004): 1317–1324. 14706563 10.1016/j.lfs.2003.09.038

[13] L. M. Newman , M. P. Lutz , and E. F. Domino , “Δ9 Tetrahydrocannabinol and Some CNS Depressants: Evidence for Cross Tolerance in the Rat,” Archives Internationales de Pharmacodynamie et de Thérapie 207, no. 2 (1974): 254–259. 4827412

[14] A. F. Hoffman and C. R. Lupica , “Direct Actions of Cannabinoids on Synaptic Transmission in the Nucleus Accumbens: A Comparison With Opioids,” Journal of Neurophysiology 85, no. 1 (2001): 72–83. 11152707 10.1152/jn.2001.85.1.72

[15] J. J. Rodríguez , K. Mackie , and V. M. Pickel , “Ultrastructural Localization of the CB1 Cannabinoid Receptor in μ‐Opioid Receptor Patches of the Rat Caudate Putamen Nucleus,” Journal of Neuroscience 21, no. 3 (2001): 823–833. 11157068 10.1523/JNEUROSCI.21-03-00823.2001 PMC6762333

[16] T. Ahmad and S. R. Laviolette , “Cannabinoid Reward and Aversion Effects in the Posterior Ventral Tegmental Area Are Mediated Through Dissociable Opiate Receptor Subtypes and Separate Amygdalar and Accumbal Dopamine Receptor Substrates,” Psychopharmacology 234 (2017): 2325–2336. 28669034 10.1007/s00213-017-4669-7

[17] J. L. Scavone , R. C. Sterling , and E. J. Van Bockstaele , “Cannabinoid and Opioid Interactions: Implications for Opiate Dependence and Withdrawal,” Neuroscience 248 (2013): 637–654. 23624062 10.1016/j.neuroscience.2013.04.034 PMC3742578

[18] L. H. Parsons and Y. L. Hurd , “Endocannabinoid Signalling in Reward and Addiction,” Nature Reviews. Neuroscience 16, no. 10 (2015): 579–594. 26373473 10.1038/nrn4004 PMC4652927

[19] S. H. Lee , M. Ledri , B. Tóth , et al., “Multiple Forms of Endocannabinoid and Endovanilloid Signaling Regulate the Tonic Control of GABA Release,” Journal of Neuroscience 35, no. 27 (2015): 10039–10057. 26157003 10.1523/JNEUROSCI.4112-14.2015 PMC4495235

[20] I. Katona , G. M. Urbán , M. Wallace , et al., “Molecular Composition of the Endocannabinoid System at Glutamatergic Synapses,” Journal of Neuroscience 26, no. 21 (2006): 5628–5637, 10.1523/JNEUROSCI.0309-06.2006. 16723519 PMC1698282

[21] D. P. Covey , Y. Mateo , D. Sulzer , J. F. Cheer , and D. M. Lovinger , “Endocannabinoid Modulation of Dopamine Neurotransmission,” Neuropharmacology 124 (2017): 52–61. 28450060 10.1016/j.neuropharm.2017.04.033 PMC5608040

[22] A. W. Zuardi , J. A. de Souza Crippa , J. E. C. Hallak , A. C. Campos , and F. S. Guimarães , “Chapter e13 ‐ The Anxiolytic Effects of Cannabidiol (CBD),” in Handbook of Cannabis and Related Pathologies, ed. V. R. Preedy (Academic Press, 2017): e131–e139, https://www.sciencedirect.com/science/article/pii/B9780128007563000971.

[23] T. C. Spinella , S. H. Stewart , J. Naugler , I. Yakovenko , and S. P. Barrett , “Evaluating Cannabidiol (CBD) Expectancy Effects on Acute Stress and Anxiety in Healthy Adults: A Randomized Crossover Study,” Psychopharmacology 238 (2021): 1965–1977. 33813611 10.1007/s00213-021-05823-w PMC8233292

[24] E. L. Martin , J. C. Strickland , N. J. Schlienz , et al., “Antidepressant and Anxiolytic Effects of Medicinal Cannabis Use in an Observational Trial,” Frontiers in Psychiatry 12 (2021): 729800. 34566726 10.3389/fpsyt.2021.729800 PMC8458732

[25] N. Stella , “THC and CBD: Similarities and Differences Between Siblings,” Neuron 111, no. 3 (2023): 302–327. 36638804 10.1016/j.neuron.2022.12.022 PMC9898277

[26] K. Singh , B. Bhushan , D. K. Chanchal , et al., “Emerging Therapeutic Potential of Cannabidiol (CBD) in Neurological Disorders: A Comprehensive Review,” Behavioural Neurology 2023, no. 1 (2023): 1–17. 10.1155/2023/8825358 PMC10586905 37868743

[27] D. L. de Almeida and L. A. Devi , “Diversity of Molecular Targets and Signaling Pathways for CBD,” Pharmacology Research & Perspectives 8, no. 6 (2020): e00682. 33169541 10.1002/prp2.682 PMC7652785

[28] A. H. Lichtman , S. M. Sheikh , H. H. Loh , and B. R. Martin , “Opioid and Cannabinoid Modulation of Precipitated Withdrawal in Δ9‐Tetrahydrocannabinol and Morphine‐Dependent Mice,” Journal of Pharmacology and Experimental Therapeutics 298, no. 3 (2001): 1007–1014, 10.1016/S0022-3565(24)29469-6. 11504797

[29] T. Yamaguchi , Y. Hagiwara , H. Tanaka , et al., “Endogenous Cannabinoid, 2‐Arachidonoylglycerol, Attenuates Naloxone‐Precipitated Withdrawal Signs in Morphine‐Dependent Mice,” Brain Research 909, no. 1–2 (2001): 121–126. 11478928 10.1016/s0006-8993(01)02655-5

[30] J. L. Wilkerson , S. Ghosh , M. Mustafa , et al., “The Endocannabinoid Hydrolysis Inhibitor SA‐57: Intrinsic Antinociceptive Effects, Augmented Morphine‐Induced Antinociception, and Attenuated Heroin Seeking Behavior in Mice,” Neuropharmacology 114 (2017): 156–167. 27890602 10.1016/j.neuropharm.2016.11.015 PMC5289715

[31] M. Solinas , L. V. Panlilio , G. Tanda , A. Makriyannis , S. A. Matthews , and S. R. Goldberg , “Cannabinoid Agonists but Not Inhibitors of Endogenous Cannabinoid Transport or Metabolism Enhance the Reinforcing Efficacy of Heroin in Rats,” Neuropsychopharmacology 30, no. 11 (2005): 2046–2057. 15870833 10.1038/sj.npp.1300754

[32] M. R. Lofwall , S. Babalonis , P. A. Nuzzo , S. C. Elayi , and S. L. Walsh , “Opioid Withdrawal Suppression Efficacy of Oral Dronabinol in Opioid Dependent Humans,” Drug and Alcohol Dependence 164 (2016): 143–150. 27234658 10.1016/j.drugalcdep.2016.05.002 PMC4910823

[33] G. P. A. Costa , J. C. Nunes , D. L. Heringer , A. Anand , and J. P. De Aquino , “The Impact of Cannabis on Non‐Medical Opioid Use Among Individuals Receiving Pharmacotherapies for Opioid Use Disorder: A Systematic Review and Meta‐Analysis of Longitudinal Studies,” American Journal of Drug and Alcohol Abuse 50, no. 1 (2024): 12–26. 38225727 10.1080/00952990.2023.2287406

[34] V. Golub and D. S. Reddy , “Cannabidiol Therapy for Refractory Epilepsy and Seizure Disorders,” in Cannabinoids and Neuropsychiatric Disorders. (Springer International Publishing, 2021), 93–110. 10.1007/978-3-030-57369-0_7 33332006

[35] R. Spanagel , “Cannabinoids and the Endocannabinoid System in Reward Processing and Addiction: From Mechanisms to Interventions,” Dialogues in Clinical Neuroscience 22, no. 3 (2020): 241–250. 33162767 10.31887/DCNS.2020.22.3/rspanagel PMC7605022

[36] Clarivate , EndNote (Clarivate, 2013).

[37] Library YUHCHWM. Reference Deduplicator [Internet], (2021), https://library.medicine.yale.edu/reference‐deduplicator.

[38] Y. L. Hurd , S. Spriggs , J. Alishayev , et al., “Cannabidiol for the Reduction of Cue‐Induced Craving and Anxiety in Drug‐Abstinent Individuals With Heroin Use Disorder: A Double‐Blind Randomized Placebo‐Controlled Trial,” American Journal of Psychiatry 176, no. 11 (2019): 911–922. 31109198 10.1176/appi.ajp.2019.18101191

[39] J. Suzuki , B. Martin , S. Prostko , P. R. Chai , and R. D. Weiss , “Cannabidiol Effect on Cue‐Induced Craving for Individuals With Opioid Use Disorder Treated With Buprenorphine: A Small Proof‐of‐Concept Open‐Label Study,” Integrative Medicine Reports. 1, no. 1 (2022): 157–163. 36105269 10.1089/imr.2022.0070 PMC9462449

[40] J. Suzuki , S. Prostko , V. Szpak , et al., “Impact of Cannabidiol on Reward‐and Stress‐Related Neurocognitive Processes Among Individuals With Opioid Use Disorder: A Pilot, Double‐Blind, Placebo‐Controlled, Randomized Cross‐Over Trial,” Frontiers in Psychiatry 14 (2023): 1155984. 37065899 10.3389/fpsyt.2023.1155984 PMC10098189

[41] A. F. Manini , G. Yiannoulos , M. M. Bergamaschi , et al., “Safety and Pharmacokinetics of Oral Cannabidiol When Administered Concomitantly With Intravenous Fentanyl in Humans,” Journal of Addiction Medicine 9, no. 3 (2015): 204–210. 25748562 10.1097/ADM.0000000000000118 PMC4449284

[42] H. N. Bhargava , “Effect of Some Cannabinoids on Naloxone‐Precipitated Abstinence in Morphine‐Dependent Mice,” Psychopharmacology 49 (1976): 267–270. 826944 10.1007/BF00426828

[43] R. L. Scicluna , B. B. Wilson , S. H. Thelaus , J. C. Arnold , I. S. McGregor , and M. T. Bowen , “Cannabidiol Reduced the Severity of Gastrointestinal Symptoms of Opioid Withdrawal in Male and Female Mice,” Cannabis and Cannabinoid Research 9, no. 2 (2024): 547–560. 36577048 10.1089/can.2022.0036

[44] G. B. Chesher and D. M. Jackson , “The Quasi‐Morphine Withdrawal Syndrome: Effect of Cannabinol, Cannabidiol and Tetrahydrocannabinol,” Pharmacology, Biochemistry and Behavior 23, no. 1 (1985): 13–15. 2994117 10.1016/0091-3057(85)90122-4

[45] B. Hine , E. Friedman , M. Torrelio , and S. Gershon , “Morphine‐Dependent Rats: Blockade of Precipitated Abstinence by Tetrahydrocannabinol,” Science (1979) 187, no. 4175 (1975): 443–445. 10.1126/science.1167428 1167428

[46] B. Hine , M. Torrelio , and S. Gershon , “Interactions Between Cannabidiol and Δ9‐THC During Abstinence in Morphine‐Dependent Rats,” Life Sciences 17, no. 6 (1975): 851–857. 1238886 10.1016/0024-3205(75)90435-x

[47] C. R. de Carvalho and R. N. Takahashi , “Cannabidiol Disrupts the Reconsolidation of Contextual Drug‐Associated Memories in Wistar Rats,” Addiction Biology 22, no. 3 (2017): 742–751. 26833888 10.1111/adb.12366

[48] J. R. Markos , H. M. Harris , W. Gul , M. A. ElSohly , and K. J. Sufka , “Effects of Cannabidiol on Morphine Conditioned Place Preference in Mice,” Planta Medica 84, no. 4 (2018): 221–224. 28793355 10.1055/s-0043-117838

[49] H. M. Harris , W. Gul , M. A. Elsohly , and K. J. Sufka , “Differential Effects of Cannabidiol and a Novel Cannabidiol Analog on Oxycodone Place Preference and Analgesia in Mice: An Opioid Abuse Deterrent With Analgesic Properties,” Cannabis and Cannabinoid Research 7, no. 6 (2022): 804–813. 34962133 10.1089/can.2021.0050 PMC9784596

[50] A. J. Souza , F. S. Guimarães , and F. V. Gomes , “Cannabidiol Attenuates the Expression of Conditioned Place Aversion Induced by Naloxone‐Precipitated Morphine Withdrawal Through the Activation of 5‐HT1A Receptors,” Behavioural Brain Research 450 (2023): 114504. 37209879 10.1016/j.bbr.2023.114504

[51] Y. Ren , J. Whittard , A. Higuera‐Matas , C. V. Morris , and Y. L. Hurd , “Cannabidiol, a Nonpsychotropic Component of Cannabis, Inhibits Cue‐Induced Heroin Seeking and Normalizes Discrete Mesolimbic Neuronal Disturbances,” Journal of Neuroscience 29, no. 47 (2009): 14764–14769. 19940171 10.1523/JNEUROSCI.4291-09.2009 PMC2829756

[52] R. Hudson , J. Renard , C. Norris , W. J. Rushlow , and S. R. Laviolette , “Cannabidiol Counteracts the Psychotropic Side‐Effects of δ‐9‐Tetrahydrocannabinol in the Ventral Hippocampus Through Bidirectional Control of erk1–2 Phosphorylation,” Journal of Neuroscience 39, no. 44 (2019): 8762–8777. 31570536 10.1523/JNEUROSCI.0708-19.2019 PMC6820200

[53] F. Navarrete , A. Gasparyan , and J. Manzanares , “CBD‐Mediated Regulation of Heroin Withdrawal‐Induced Behavioural and Molecular Changes in Mice,” Addiction Biology 27, no. 2 (2022): e13150. 35229949 10.1111/adb.13150

[54] S. Jin , C. Lin , Y. Wang , et al., “Cannabidiol Analogue CIAC001 for the Treatment of Morphine‐Induced Addiction by Targeting PKM2,” Journal of Medicinal Chemistry 66, no. 16 (2023): 11498–11516, 10.1021/acs.jmedchem.3c01029. 37531582

[55] M. T. Rivera‐Garcia , R. M. Rose , and A. R. Wilson‐Poe , “High‐CBD Cannabis Vapor Attenuates Opioid Reward and Partially Modulates Nociception in Female Rats,” Addiction Neuroscience 5 (2023): 100050. 36937502 10.1016/j.addicn.2022.100050 PMC10019487

[56] V. Katsidoni , I. Anagnostou , and G. Panagis , “Cannabidiol Inhibits the Reward‐Facilitating Effect of Morphine: Involvement of 5‐HT1A Receptors in the Dorsal Raphe Nucleus,” Addiction Biology 18, no. 2 (2013): 286–296. 22862835 10.1111/j.1369-1600.2012.00483.x

[57] L. M. Carey , D. R. Maguire , and C. P. France , “Effects of Δ^9^‐Tetrahydrocannabinol (THC), Cannabidiol (CBD), and THC/CBD Mixtures on Fentanyl Versus Food Choice in Rhesus Monkeys,” Drug and Alcohol Dependence 244 (2023): 109787. 36753805 10.1016/j.drugalcdep.2023.109787 PMC10697211

[58] E. Chesney , D. Oliver , A. Green , et al., “Adverse Effects of Cannabidiol: A Systematic Review and Meta‐Analysis of Randomized Clinical Trials,” Neuropsychopharmacology 45, no. 11 (2020): 1799–1806. 32268347 10.1038/s41386-020-0667-2 PMC7608221

[59] L. A. Lo , A. Christiansen , L. Eadie , et al., “Cannabidiol‐Associated Hepatotoxicity: A Systematic Review and Meta‐Analysis,” Journal of Internal Medicine 293, no. 6 (2023): 724–752. 36912195 10.1111/joim.13627

[60] J. W. Skelley , C. M. Deas , Z. Curren , and J. Ennis , “Use of Cannabidiol in Anxiety and Anxiety‐Related Disorders,” Journal of the American Pharmacists Association 60, no. 1 (2020): 253–261. 31866386 10.1016/j.japh.2019.11.008

[61] A. W. Zuardi , F. S. Guimarães , and A. C. Moreira , “Effect of Cannabidiol on Plasma Prolactin, Growth Hormone and Cortisol in Human Volunteers,” Brazilian Journal of Medical and Biological Research 26, no. 2 (1993): 213–217. 8257923

[62] M. Nardone , C. P. Cheung , R. E. Baker , et al., “Inhalation of THC‐Containing Cannabis Selectively Diminishes Cardiac Autonomic Function in Humans,” Clinical Autonomic Research 33, no. 6 (2023): 919–922. 37907708 10.1007/s10286-023-00993-3

[63] J. A. Crippa , A. W. Zuardi , G. E. Garrido , et al., “Effects of Cannabidiol (CBD) on Regional Cerebral Blood Flow,” Neuropsychopharmacology 29, no. 2 (2004): 417–426. 14583744 10.1038/sj.npp.1300340

[64] B. D. Kiluk , B. A. Kleykamp , S. D. Comer , et al., “Clinical Trial Design Challenges and Opportunities for Emerging Treatments for Opioid Use Disorder: A Review,” JAMA Psychiatry 80, no. 1 (2023): 84–92. 36449315 10.1001/jamapsychiatry.2022.4020 PMC10297827

[65] Y. L. Hurd , M. Yoon , A. F. Manini , et al., “Early Phase in the Development of Cannabidiol as a Treatment for Addiction: Opioid Relapse Takes Initial Center Stage,” Neurotherapeutics 12, no. 4 (2015): 807–815. 26269227 10.1007/s13311-015-0373-7 PMC4604178

[66] C. K. Nielsen , F. B. Ross , S. Lotfipour , K. S. Saini , S. R. Edwards , and M. T. Smith , “Oxycodone and Morphine Have Distinctly Different Pharmacological Profiles: Radioligand Binding and Behavioural Studies in Two Rat Models of Neuropathic Pain,” Pain 132, no. 3 (2007): 289–300. 17467904 10.1016/j.pain.2007.03.022

[67] A. E. Olesen , C. Staahl , L. Arendt‐Nielsen , and A. M. Drewes , “Different Effects of Morphine and Oxycodone in Experimentally Evoked Hyperalgesia: A Human Translational Study,” British Journal of Clinical Pharmacology 70, no. 2 (2010 Aug): 189–200. 20653672 10.1111/j.1365-2125.2010.03700.x PMC2911549

[68] C. J. A. Morgan , G. Schafer , T. P. Freeman , and H. V. Curran , “Impact of Cannabidiol on the Acute Memory and Psychotomimetic Effects of Smoked Cannabis: Naturalistic Study,” British Journal of Psychiatry 197, no. 4 (2010): 285–290. 10.1192/bjp.bp.110.077503 20884951

[69] K. Mackie , “Cannabinoid Receptors: Where They Are and What They Do,” Journal of Neuroendocrinology 20 (2008): 10–14. 18426493 10.1111/j.1365-2826.2008.01671.x

[70] R. Pertwee , “The Diverse CB1 and CB2 Receptor Pharmacology of Three Plant Cannabinoids: Δ9‐Tetrahydrocannabinol, Cannabidiol and Δ9‐Tetrahydrocannabivarin,” British Journal of Pharmacology 153, no. 2 (2008): 199–215. 17828291 10.1038/sj.bjp.0707442 PMC2219532

[71] T. Bisogno , L. Hanuš , L. De Petrocellis , et al., “Molecular Targets for Cannabidiol and Its Synthetic Analogues: Effect on Vanilloid VR1 Receptors and on the Cellular Uptake and Enzymatic Hydrolysis of Anandamide,” British Journal of Pharmacology 134, no. 4 (2001): 845–852. 11606325 10.1038/sj.bjp.0704327 PMC1573017

[72] M. Kathmann , K. Flau , A. Redmer , C. Tränkle , and E. Schlicker , “Cannabidiol Is an Allosteric Modulator at mu‐and delta‐Opioid Receptors,” Naunyn‐Schmiedeberg's Archives of Pharmacology 372 (2006): 354–361. 16489449 10.1007/s00210-006-0033-x

[73] D. Vigano , T. Rubino , and D. Parolaro , “Molecular and Cellular Basis of Cannabinoid and Opioid Interactions,” Pharmacology, Biochemistry, and Behavior 81, no. 2 (2005): 360–368. 15927245 10.1016/j.pbb.2005.01.021

[74] G. F. Koob , “Drugs of Abuse: Anatomy, Pharmacology and Function of Reward Pathways,” Trends in Pharmacological Sciences 13 (1992): 177–184. 1604710 10.1016/0165-6147(92)90060-j

[75] E. B. Russo , A. Burnett , B. Hall , and K. K. Parker , “Agonistic Properties of Cannabidiol at 5‐HT1a Receptors,” Neurochemical Research 30 (2005): 1037–1043. 16258853 10.1007/s11064-005-6978-1

[76] J. Renard , C. Norris , W. Rushlow , and S. R. Laviolette , “Neuronal and Molecular Effects of Cannabidiol on the Mesolimbic Dopamine System: Implications for Novel Schizophrenia Treatments,” Neuroscience and Biobehavioral Reviews 75 (2017): 157–165. 28185872 10.1016/j.neubiorev.2017.02.006

[77] C. M. Pretzsch , J. Freyberg , B. Voinescu , et al., “Effects of Cannabidiol on Brain Excitation and Inhibition Systems; A Randomised Placebo‐Controlled Single Dose Trial During Magnetic Resonance Spectroscopy in Adults With and Without Autism Spectrum Disorder,” Neuropsychopharmacology 44, no. 8 (2019): 1398–1405. 30758329 10.1038/s41386-019-0333-8 PMC6784992

