# Supplementary material for: The Potential Use of Cannabidiol in the Treatment of Opioid Use Disorder: A Systematic Review
Source: Addict Biol. 2025 May 25;30(5):e70047. doi: 10.1111/adb.70047 (PMC12104536; doi:10.1111/adb.70047)
Supplement: Supplementary file 1 — Table S1. The search strategy of databases. [file ADB-30-e70047-s001.docx]

**Table S1.** Search strategy of databases.

| **Database** | **Search** | **Query** | **Results (No.)** |
| --- | --- | --- | --- |
| Ovid MEDLINE | #1 | Cannabidiol/ or (Cannabidiol or CBD).tw,kw. | 536 |
|  | #2 | exp Opioid-Related Disorders/ or Opiate Substitution Treatment/ or exp buprenorphine/ or exp naloxone/ or Methadone/ or Fentanyl/ or (analgesic* or buprenorphine or codeine or dihydromorphine or diamorphine or fentanyl or heroin or hydrocodone or hydromorphone or levorphanol or meperidine or methadone or morfin* or morphin* or naloxone or naltrexone or narcotic* or opiate* or opioid* or opium or oxycodone or oxycontin or oxymorphone or propoxyphene or tramadol).tw,kf. |  |
|  | #3 | #1 AND #2 |  |
| Embase (Ovid) | #1 | Cannabidiol/ or (Cannabidiol or CBD).tw,kw. | 1177 |
|  | #2 | exp narcotic dependence/ or opiate overdose/ or opiate substitution treatment/ or buprenorphine/ or naloxone/ or methadone/ or fentanyl/ or (analgesic* or buprenorphine or codeine or dihydromorphine or diamorphine or fentanyl or heroin or hydrocodone or hydromorphone or levorphanol or meperidine or methadone or morfin* or morphin* or naloxone or naltrexone or narcotic* or opiate* or opioid* or opium or oxycodone or oxycontin or oxymorphone or propoxyphene or tramadol).tw,kw. |  |
|  | #3 | #1 AND #2 |  |
| APA PsycInfo (Ovid) | #1 | Cannabidiol/ or (Cannabidiol or CBD).mp. | 147 |
|  | #2 | exp "opioid use disorder"/ or exp maintenance therapy/ or exp medication-assisted treatment/ or buprenorphine/ or naloxone/ or methadone/ or fentanyl/ or (analgesic* or buprenorphine or codeine or dihydromorphine or diamorphine or fentanyl or heroin or hydrocodone or hydromorphone or levorphanol or meperidine or methadone or morfin* or morphin* or naloxone or naltrexone or narcotic* or opiate* or opioid* or opium or oxycodone or oxycontin or oxymorphone or propoxyphene or tramadol).mp. |  |
|  | #3 | #1 AND #2 |  |
| Web of Science Core Collection (Clarivate) | #1 | TS=(Cannabidiol or CBD) | 604 |
|  | #2 | TS=(analgesic* or buprenorphine or codeine or dihydromorphine or diamorphine or fentanyl or heroin or hydrocodone or hydromorphone or levorphanol or meperidine or methadone or morfin* or morphin* or naloxone or naltrexone or narcotic* or opiate* or opioid* or opium or oxycodone or oxycontin or oxymorphone or propoxyphene or tramadol) |  |
|  | #3 | #1 AND #2 |  |
| Scopus (Elsevier) | #1 | TITLE-ABS ( cannabidiol OR cbd ) | 571 |
|  | #2 | TITLE-ABS ( analgesic* OR buprenorphine OR codeine OR dihydromorphine OR diamorphine OR fentanyl OR heroin OR hydrocodone OR hydromorphone OR levorphanol OR meperidine OR methadone OR morfin* OR morphin* OR naloxone OR naltrexone OR narcotic* OR opiate* OR opioid* OR opium OR oxycodone OR oxycontin OR oxymorphone OR propoxyphene OR tramadol ) |  |
|  | #3 | #1 AND #2 |  |
| Cochrane (Wiley) (CDSR+CENTRAL) | #1 | ((Cannabidiol or CBD)):ti,ab,kw | 3+186 |
|  | #2 | ((analgesic* or buprenorphine or codeine or dihydromorphine or diamorphine or fentanyl or heroin or hydrocodone or hydromorphone or levorphanol or meperidine or methadone or morfin* or morphin* or naloxone or naltrexone or narcotic* or opiate* or opioid* or opium or oxycodone or oxycontin or oxymorphone or propoxyphene or tramadol)):ti,ab,kw |  |
|  | #3 | #1 AND #2 |  |
